# Enhancing SARS-CoV-2 Surveillance through Regular Genomic Sequencing in Spain: The RELECOV Network

**DOI:** 10.3390/ijms24108573

**Published:** 2023-05-10

**Authors:** Sonia Vázquez-Morón, María Iglesias-Caballero, José Antonio Lepe, Federico Garcia, Santiago Melón, José M. Marimon, Darío García de Viedma, Maria Dolores Folgueira, Juan Carlos Galán, Carla López-Causapé, Rafael Benito-Ruesca, Julia Alcoba-Florez, Fernando Gonzalez Candelas, María de Toro, Miguel Fajardo, Carmen Ezpeleta, Fernando Lázaro, Sonia Pérez Castro, Isabel Cuesta, Angel Zaballos, Francisco Pozo, Inmaculada Casas, on behalf of RELECOV Network Members

**Affiliations:** 1 Respiratory Viruses and Influenza Unit, National Centre for Microbiology, Instituto de Salud Carlos III, 28222 Majadahonda, Spain; 2 CIBER de Epidemiología y Salud Pública (CIBERESP), ISCIII, 28029 Madrid, Spain; 3 Microbiology Service, Hospital Universitario Virgen del Rocio, 41013 Sevilla, Spain; 4 Microbiology Service, Hospital Universitario San Cecilio, Instituto de Investigación Biosanitaria Ibs. Granada, 18016 Granada, Spain; 5 CIBER de Enfermedades Infecciosas (CIBERINFEC), ISCIII, 28029 Madrid, Spain; 6 Microbiology Service, Hospital Universitario Central de Asturias, 33011 Oviedo, Spain; 7 Microbiology Service, Instituto de Investigación Sanitaria Biodonostia, Hospital Universitario Donostia, 20014 Donostia-San Sebastian, Spain; 8 Microbiology Service, Hospital General Universitario Gregorio Marañón, Instituto de Investigación Sanitaria Gregorio Marañón, 28007 Madrid, Spain; 9 Microbiology Department, Hospital Universitario 12 de Octubre, Biomedical Research Institute imas12, 28041 Madrid, Spain; 10 Department of Medicine, School of Medicine, Universidad Complutense, 28040 Madrid, Spain; 11 Microbiology Service, Hospital Universitario Ramón y Cajal, Instituto Ramón y Cajal de Investigación Sanitaria (IRYCIS), 28034 Madrid, Spain; 12 Microbiology Service, Hospital Universitario Son Espases, 07120 Palma de Mallorca, Spain; 13 Microbiology Service, Hospital Clínico Universitario Lozano Blesa, Departamento de Microbiología, Facultad de Medicina, Instituto de Investigación Sanitaria de Aragón, Universidad de Zaragoza, 50009 Zaragoza, Spain; 14 Microbiology Service, Hospital Universitario Ntra. Sra de Candelaria, 38010 Santa Cruz de Tenerife, Spain; 15 Joint Research Unit Infection and Public Health FISABIO-University of Valencia, Institute for Integrative Systems Biology (I2SysBio), 46020 Valencia, Spain; 16 Plataforma de Genómica y Bioinformática, Centro de Investigación Biomédica de La Rioja (CIBIR), 26006 Logroño, Spain; 17 Microbiology Service, Hospital Universitario de Badajoz, 06080 Badajoz, Spain; 18 Complejo Hospitalario de Navarra and Navarra De Servicios Y Tecnologías S A (NASERTIC), 31008 Pamplona, Spain; 19 Microbiology Service, Hospital Universitario La Paz, 28046 Madrid, Spain; 20 Microbiology Service, Complexo Hospitalario Universitario de Vigo, 36204 Vigo, Spain; 21 Bioinformatics Unit, Unidades Centrales Científico Técnicas, Instituto de Salud Carlos III, 28222 Majadahonda, Spain; 22 Genomics Unit, Unidades Centrales Científico Técnicas, Instituto de Salud Carlos III, 28222 Majadahonda, Spain

**Keywords:** SARS-CoV-2, genomic surveillance Spain, laboratory network RELECOV, VOCs, lineages, national QCA, mutation, phylogeny

## Abstract

Millions of SARS-CoV-2 whole genome sequences have been generated to date. However, good quality data and adequate surveillance systems are required to contribute to meaningful surveillance in public health. In this context, the network of Spanish laboratories for coronavirus (RELECOV) was created with the main goal of promoting actions to speed up the detection, analyses, and evaluation of SARS-CoV-2 at a national level, partially structured and financed by an ECDC-HERA-Incubator action (ECDC/GRANT/2021/024). A SARS-CoV-2 sequencing quality control assessment (QCA) was developed to evaluate the network’s technical capacity. QCA full panel results showed a lower hit rate for lineage assignment compared to that obtained for variants. Genomic data comprising 48,578 viral genomes were studied and evaluated to monitor SARS-CoV-2. The developed network actions showed a 36% increase in sharing viral sequences. In addition, analysis of lineage/sublineage-defining mutations to track the virus showed characteristic mutation profiles for the Delta and Omicron variants. Further, phylogenetic analyses strongly correlated with different variant clusters, obtaining a robust reference tree. The RELECOV network has made it possible to improve and enhance the genomic surveillance of SARS-CoV-2 in Spain. It has provided and evaluated genomic tools for viral genome monitoring and characterization that make it possible to increase knowledge efficiently and quickly, promoting the genomic surveillance of SARS-CoV-2 in Spain.

## 1. Introduction

On 30 January 2020, the World Health Organization (WHO) declared the 2019 coronavirus disease (COVID-19) outbreak a public health emergency of international concern (PHEIC) [1]. One week later, the WHO recommended a surveillance approach based on, or similar to, the Global Influenza Surveillance and Response System (GISRS) that facilitates less resource-intensive monitoring [2]. Thus, from the beginning of the pandemic caused by SARS-CoV-2 [3,4,5], the frantic race to sequence this virus began, promoted by rapid technological development. This has led to the description of millions of genome sequences to date from the first SARS-CoV-2 complete genome published from a patient with severe respiratory syndrome on 26 December 2019 [4].

This large amount of information on the SARS-CoV-2 genome has aroused different nomenclature systems, genome repositories, and web servers allowing the exchange of molecular information as well as its analysis. Currently, three nomenclature systems, with their own scientific approaches, are used to classify and track SARS-CoV-2. The Global Initiative on Sharing All Influenza Data (GISAID) offers EpiCoV^TM^ that includes a data repository, nomenclature system, and many tools for viral analysis [6]. Additionally, the NEXSTRAIN web server allows SARS-CoV-2 genome analyses and visualizations of the data [7]. In addition, the third nomenclature system is known as Pangolin which was developed for the implementation of a dynamic nomenclature for SARS-CoV-2. This has been described by Rambaut et al. and is known as “Pango lineage” [8,9]. This tool is used to assign lineages based on complete virus genome sequences, date of detection, and geographical location, allowing us to delve into the viral genome, and its evolution and transmission. However, this system has generated more than a thousand lineages, which constitute a great challenge for their follow-up in public health. In this sense, in May 2021, the WHO announced the assignment of simple names, easy to say and remember for the variants designated as of concern (VOCs) or interest (VOIs), using the Greek alphabet and arguing to avoid stigmatization generated by the relationship between the variant and the country where it was discovered and developing a global risk-monitoring framework [10].

SARS-CoV-2 and other coronaviruses evolve rapidly through point mutations as well as recombination, specifically in the spike region which is both a recombination and a mutation hotspot in coronaviruses [11,12]. SARS-CoV-2 evolution studies estimated a substitution rate of 0.5 × 10^−3^–1.1 × 10^−3^ substitutions/site/year, corresponding to a rate of 1.3 to 2.8 substitutions/month for the whole genome, and the increases in substitution rates have been related to the emergence of new variants [13]. However, the spike region, in particular, accumulates mutations much faster than other regions. The emergence of the Omicron variant was a great example, because it accumulated an unusually high number of mutations in the spike region that was not observed previously with any other VOC [12], and led to evidence of a substantial reduction of the antibody neutralizing activity against Omicron [14].

These viral characteristics showed the need to increase molecular knowledge related to the transmission and spread of the virus that could raise concern, which is essential for public health. In this sense, not only is the massive generation of data important, but these must be of good quality and linked to the establishment of an adequate surveillance system in order to contribute to meaningful actions in public health. In this regard, the WHO and European Centre for Disease Prevention and Control (ECDC) guidance on implementing genomic SARS-CoV-2 surveillance were released in January and May 2021, respectively [15,16]. On the other hand, on 17 February 2021, the European Commission proposed immediate action to prepare Europe for the growing threat of coronavirus variants under a new plan promoted by the program “Health Emergency Preparedness and Response Authority (HERA) Incubator” [17]. In Spain, the creation of a network of Spanish laboratories (RELECOV) for genomic surveillance of SARS-CoV-2 was a priority starting from January 2021 and it was reflected in the document “Strategy for the integration of sequencing in the surveillance of SARS-CoV-2” [18]. The RELECOV network was then officially structured under the ECDC/HERA/2021/024 project “Enhancing whole genome sequencing (WGS) and/or reverse transcription polymerase chain reaction (RT-PCR), national infrastructures, and capacities to respond to the COVID-19 pandemic in Spain”. The network, composed initially of 43 member institutions, including some regional nodes with regional networks or well-defined consortia. The RELECOV’s main objectives include promoting actions to speed up SARS-CoV-2 genomic virus detection, analyses, and evaluation in Spain. RELECOV’s efforts to date have been focused on improving and enhancing the SARS-CoV-2 genomic surveillance directed at the circulating virus in Spain, through establishing national quality controls for sequencing performance and methodology implementation, implementing genomic analyses and sharing of the obtained sequences, tracking mutations in the genome and, finally, performing phylogenetic analyses to characterize the sequenced viral genomes to provide a global context to the viruses circulating in Spain. These actions are essential given the potential scenarios for the rise to new pathogens or variants, highlighting the need to continue research on coronaviruses and especially genomic surveillance [19].

## 2. Results

### 2.1. Surveillance of Virus from Sequences Deposited in GISAID by the RELECOV Network

The first six months from the structuring of the RELECOV network were evaluated by monitoring viral circulation and the progress in sequence sharing in Spain. For this purpose, three periods were established to observe network’s activity over time: two quarterly periods comprised of 05.09.2021 to 05.12.2021 and 06.12.2021 to 08.03.2022, and their corresponding semi-annual period (05.09.2021 to 08.03.2022).

RELECOV’s complete sequences deposited in GISAID in the period between 05.09.2021 and 05.12.2021 showed a spectrum of up to two hundred different lineages. The classification of the corresponding variants showed 1185 Alpha, 87 Beta, 18,272 Delta, 96 Gamma, 171 Mu, 2 Iota, 8 Omicron, 40 Lambda, 27 Eta, 1 Epsilon, 1 VUM (Variant Under Monitoring), 483 NA (Not applicable, lineages circulating before variant assignment started) 61 with an unassigned lineage, and 3 classified as recombinant (Table 1). The results showed that the Variant of Concern (VOC) with the highest circulation in Spain during this period was Delta, followed to a lesser extent by Alpha, which is consistent with the worldwide patterns at that time.

Between 06.12.2021and 08.03.2022, the virus genomes deposited in GISAID belonged to 172 different lineages, corresponding to the following variant classification: 72 Alpha, 2 Beta, 12,733 Delta, 4 Gamma, 15 Mu, 15,251 Omicron, 1 recombinant (XG), 49 NA, and 16 with an unassigned lineage (Table 2). For this period, the obtained results allowed us to monitor the co-circulation of the Omicron and Delta variants in similar proportions, as well as the quick spreading of the Omicron variant during this period.

**Figure 1:**
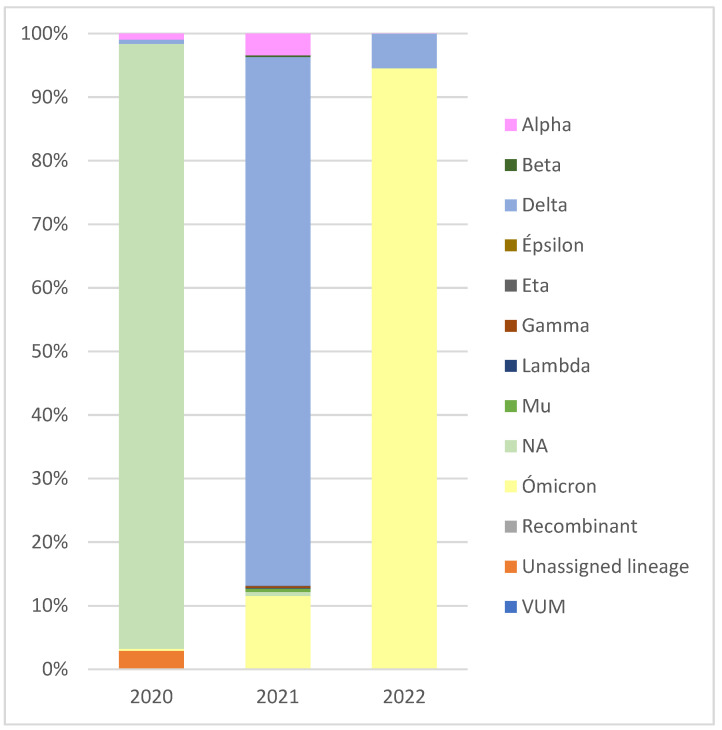
Percentage of sequences corresponding to different variants by year. SARS-CoV-2 viral sequences from Spain shared in GISAID between 05.09.2021 and 08.03.2022. NA: not applicable (lineages without variant assignment). VUM: Variant Under Monitoring.

The aggregated data for a semi-annual period (05.09.2021 to 08.03.2022) showed that viral sequences deposited in GISAID corresponded to 263 different lineages. In terms of variant classification, we found 1257 sequences belonging to Alpha, 89 Beta, 31,005 Delta, 27 Gamma, 1 Epsilon, 27 Eta, 40 Lambda, 186 Mu, 15,259 Omicron, 4 recombinant, 532 NA, 1 VUM, and 77 sequences with an unassigned lineage. The analysis also allowed us to observe sequences deposited during the study period but corresponding to prior dates, which demonstrates the network boost in data exchange. During 2020, an elevated percentage of virus circulation without variant assignment was observed since this nomenclature system was announced by the WHO in May 2021 [20]. Moreover, there was a high percentage of the Delta variant circulating in 2021 and a high percentage for the Omicron variant in the two first months of 2022 in Spain. Other variants such as Beta, Mu, Lambda, and Gamma circulated in Spain in smaller proportions during 2021 (Figure 1).

**Figure 2:**
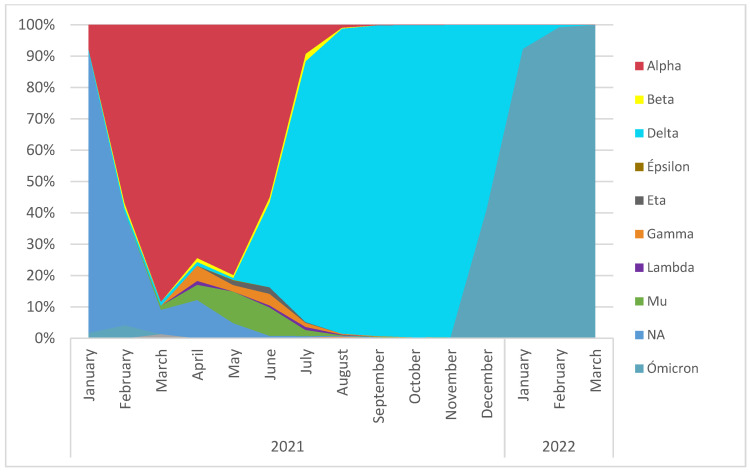
Time course of variants in Spain between 05.09.2021 and 08.03.2022 by month (sequences shared in GISAID). NA: Not applicable (lineages without variant assignment). VUM: Variant Under Monitoring.

The monthly based analysis for the semi-annual period data (05.09.2021 to 08.03.2022) showed co-circulation of the main VOCs and their replacement in Spain. These data highlight the co-circulation of Alpha and Delta variants during June and July 2021, with Delta becoming the dominant variant in August 2021. The co-circulation of Delta and Omicron variants in December 2021 was also detected, concluding with the replacement of Delta by Omicron in February 2022 (Figure 2) reflecting similar dynamics for Alpha, Delta, and Omicron time course distributions in all submitted sequences to GISAID until 15.03.2022 (Supplementary File S3 and Supplementary Figure S1).

Moreover, comparing the data by dividing the semi-annual period by quarter (05.09.2021–05.12.2021 and 06.12.2021–08.03.2022), an increase of 36% in the number of deposited viral sequences from Spain in GISAID was observed, which demonstrates data sharing improvement over time.

### 2.2. Tracking Changes in Delta and Omicron Variants

To track changes in Delta and Omicron variants, we checked all lineage-defining mutations, considered as non-synonymous substitutions or deletions that occur in >75% of sequences within a lineage or its sublineages according to their definition on the web portal outbreak.info [21].

#### 2.2.1. Delta Variant

The analyses of lineage-defining mutations corresponding to 248 Delta lineages and sublineages available until July 2022 were checked. The analyses showed a total of 441 mutations described for this set corresponding to the Delta variant, of which 14 were present in more than 98% of lineages and sublineages. These mutations were: *ORF1b* (P314L, G662S, and P1000L); *S* (T19R, T452R, T478K, D614G, and P681R); *ORF3a* (S26L); *M* (I82T); *N* (D63G, R203M, and D377Y); and *ORF8* (S84L). The mutations present in all lineages and sublineages were: T19R, T478K, and D614G in *S*, I82T in *M*, and S26L in *ORF3a*.

#### 2.2.2. Omicron Variant

The analysis for the Omicron variant included a total of 205 lineages and sublineages available until October 2022. A total of 343 mutations were present in this set, with the most frequent being: *ORF1a* (P3395H and T3255I); *ORF1b* (P314L and I1566V); *S* (G142D, G339D, S373P, S375F, S447N, T478K, E484A, Y505H, D614G, H655Y, N679K, P681H, N764K, D796Y, Q954H, and N969K), *E* (T19I); M (Q19E and A63T); *ORF8* (S84L); and *N* (P13L, R203K, and G204R). However, only the mutations in *ORF1a* (T3255I and P3395H), *S* (D614G, H655Y, N679K, D796Y, Q954H, and N969K), and *M* (A63T) were present in all lineages and sublineages belonging to the Omicron variant. These results showed that 27 mutations were present in more than 95% of lineages/sublineages belonging to the Omicron variant, but only 9 were present in all of them, and most mutations were located in the *S* gene.

In this analysis, we also observed that the Delta lineages/sublineages had three times more defined lineage mutations (441) than Omicron (161) until July 2022. We suggest that this difference might be associated with the circulation period of each variant until the moment of analysis (Delta: October 2020 to July 2022 and Omicron: November 2021 to July 2002) and could be related to the number of infections, giving rise to a greater evolution of Delta reflected in the changes established as determinants of lineages. A posterior analysis on October 2022 revealed an increase to 342 lineage-defining mutations of Omicron lineages/sublineages, confirming its evolution over time.

### 2.3. Quality Control Assessment for SARS-CoV-2 Sequencing (QCA)

The QCA of SARS-CoV-2 sequencing was performed between 22.12.2021 and 28.01.2022. In total, 37 out of the 38 RELECOV network participants (97.4%) reported their results for the quality control assessment. Obtaining and sequencing of inactivated and lyophilized SARS-CoV-2 samples was carried out and assessed by the Reference Laboratory, Respiratory Virus and Influenza Unit of the National Centre for Microbiology (CNM), Carlos III Health Institute before dispatch, in order to obtain the correct results for the assignment of lineage and variant for each sample. The sample details for the QCA are shown in Table 3.

In regard to the library preparation kits, the most used by the participants were the DNA prep Tagmentation (35.14%), Nextera XT, and SeqCovid kits (10.81% each for the latter two) on the Illumina platform which was used by 70.27% of the participants. The Ion ampliseq kit for Chef DL8 (8.10%) on the Ion Torrent platform was used by 27.03% of the participants.

**Figure 3:**
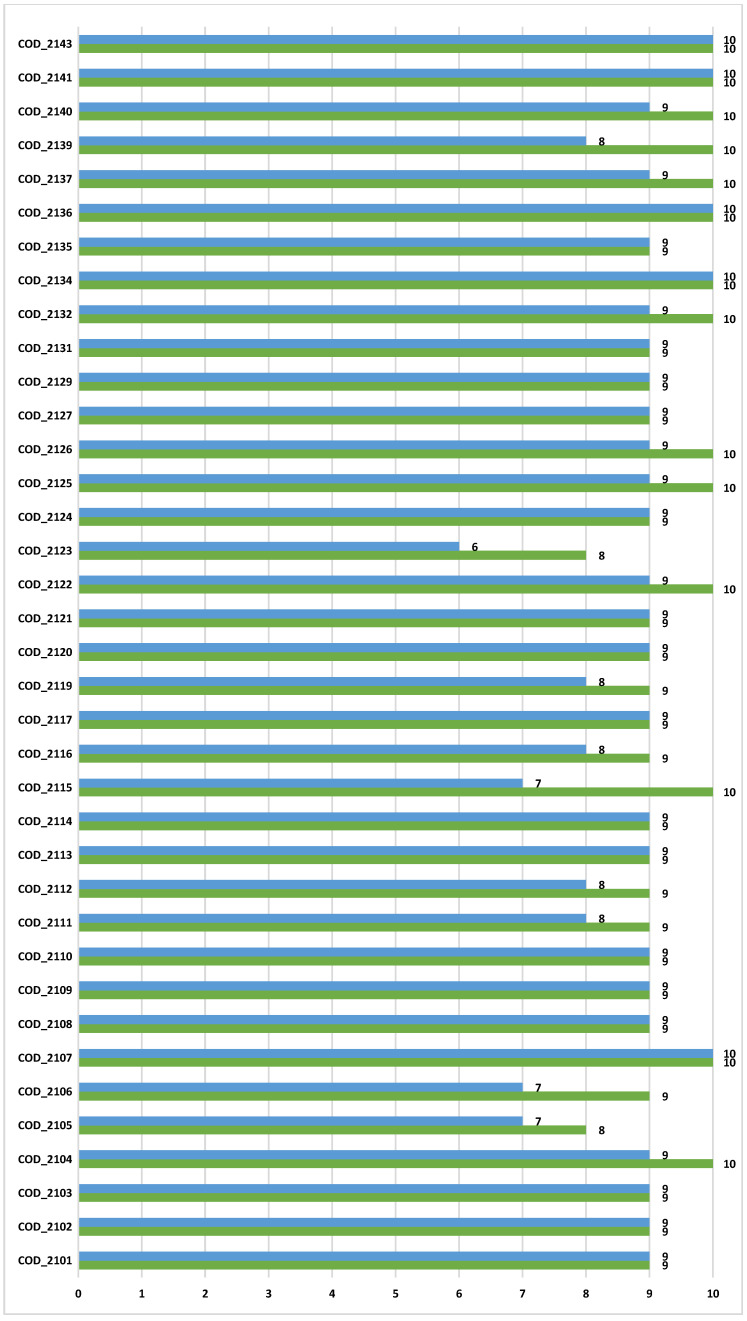
Results for variant (green) and lineage (blue) assignments in ten samples for the RELECOV network participants.

The overall results obtained by the RELECOV members, who were individually designated by a unique and random code anonymized, are shown in Figure 3.

The complete hit rates (percentage of assignment) for variant and lineage were 37.8% and 13.51%, respectively, and the correct assignment of both variant and lineage was low 13.51% (5/37). We identified a potential problem with one of the QCA samples, due to a failure in the RNA lyophilization procedure therefore, batches of tubes corresponding to this specific sample were not homogeneous. For analysis of results, hit rates were recalculated without considering that specific sample. After removing this sample, the global results generated a complete correct assignment rate of 94.59% for variant and 64.86% for lineage. In addition, performing a disaggregated analysis by QCA sample, the variant and lineage assignments were above 95% and 85%, respectively, regardless of the described problem with one sample. These results highlight the inherent complexity of comparing lineage assignments from a number of laboratories using different sequencing technologies and bioinformatic pipelines to generate the consensus SARS-CoV-2 sequences.

### 2.4. Reference SARS-CoV-2 Phylogenetic Analysis Implementation

The phylogenetic analyses were performed in order to establish a reference matrix and tree containing worldwide sequences including different lineages and variants as a tool for surveillance purposes.

**Figure 4:**
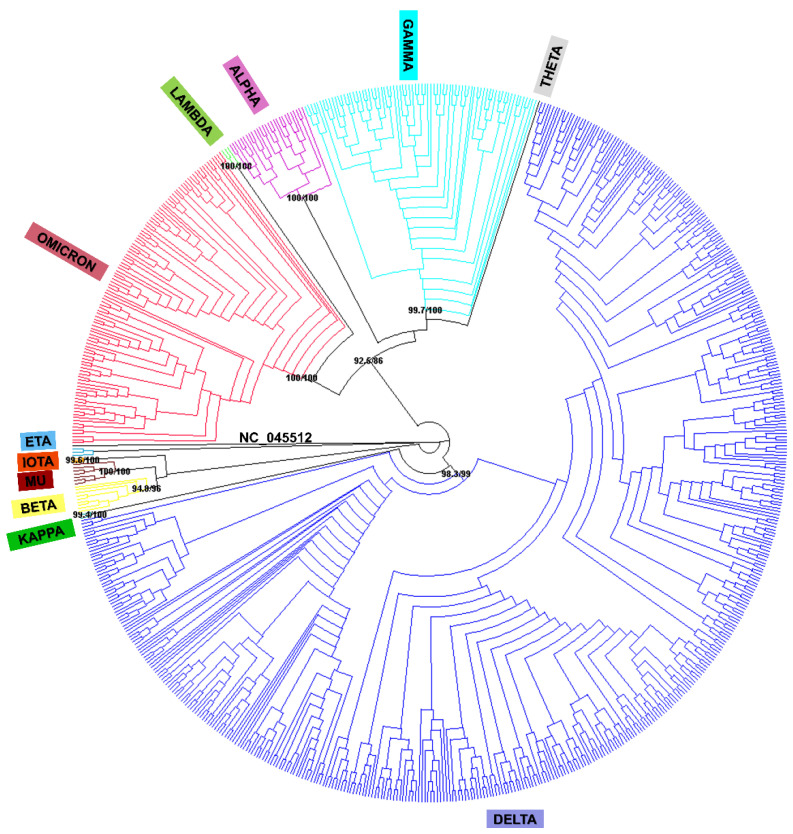
Phylogenetic tree, showed as cladogram, of the 713 worldwide (76 countries and territories) complete viral genomes retrieved from GISAID and NCBI NC_045512.2 SARS-CoV-2. Each colour represents a cluster associated with a specific variant identified by WHO. Nodes values: SH-aLRT/UFBoot (ultrafast bootstrap) supports.

**Figure 5:**
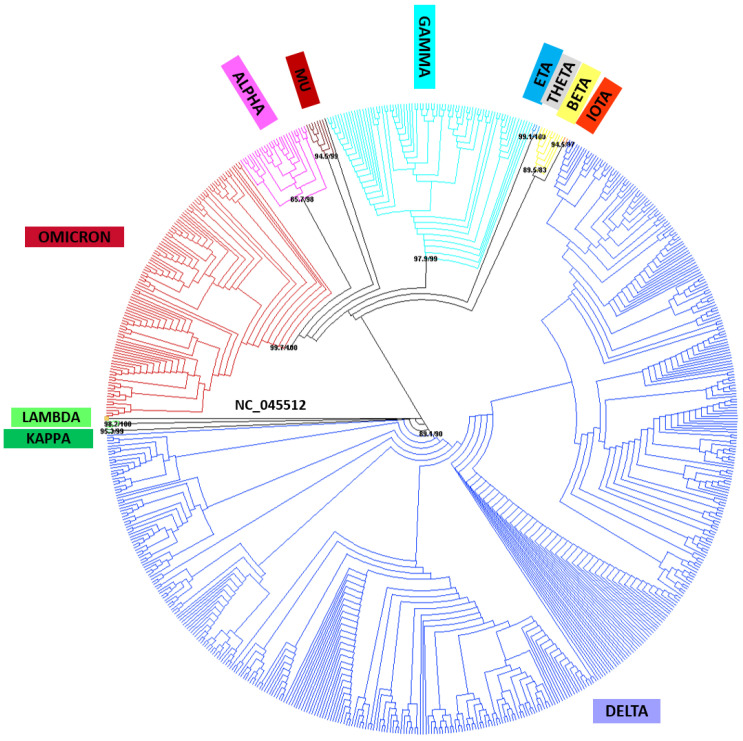
Phylogenetic tree, showed as cladogram, for the *S* gene of the 713 worldwide (76 countries and territories) sequences retrieved from GISAID and NCBI NC_045512.2 SARS-CoV-2 reference. Each colour represents a cluster associated with specific variants identified by WHO. Nodes values: SH-aLRT/UFBoot (ultrafast bootstrap) supports.

A phylogenetic analysis was carried out using FastTree [22] and IQ-TREE 2 [23] from the FASTA files corresponding to 713 worldwide (76 countries and territories) viral sequences retrieved from GISAID using the NCBI Sequence NC_045512.2 as the reference genome for SARS-CoV-2. This analysis revealed the same clusters described by the WHO for variant assignments comprising different lineages and sublineages (Figure 4). An additional analysis using the sequence of the *S* gene (Figure 5) showed that variant clusters were consistent in both trees. As expected, viral evolution was better resolved using the complete viral genome for this analysis. However, the information provided by the *S* gene must be taken into consideration in view of the results obtained for variant classifications.

## 3. Discussion

The main goal in public health is the prevention of disease and promotion of health. For this purpose, different methods have been used to track the progression of diseases, locate outbreaks, and establish containment strategies. In recent years, genomic methods have given a great boost to the surveillance of infectious diseases, allowing us to track their spread in the population, their aetiology, as well as their resistance to drugs and vaccines, and becoming a priority for the surveillance of infectious diseases even before the SARS-CoV-2 pandemic [24].

Historically, genomic surveillance has only been routinely performed in a few countries due to the complexity and cost of the technology. However, these barriers have been changing during the COVID-19 pandemic, and genomic information on pathogens has been seen as crucial for public health decision making [25]. Consequently, in 2022, the WHO launched a strategy to strengthen and expand genomic surveillance worldwide [18]. At present, this information is highly relevant, since it provides knowledge that allows the identification of the pathogen, its circulation, and makes it possible to plan a response to the disease. Therefore, the establishment of genomic surveillance networks play a key role as tool that helps drive rapid public health decisions.

However, as far as we know, there is little data about the establishment of genomic surveillance networks in the SARS-CoV-2 pandemic context that have been published [26,27], the first being The Coronavirus Disease 2019 (COVID-19) Genomics UK Consortium (COG-UK) launched in May 2020, which would lay the groundwork for genomics to serve as core outbreak tracking tool in the future [28]. In Spain, during the initial pandemic situation, the SeqCOVID consortium contributed to the monitoring and study of the evolution of the epidemic [29]. The RELECOV network of laboratories was created to consolidate a national network to enhance the SARS-CoV-2 genomic surveillance, becoming one of the countries that had performed a high level of routine genomic surveillance and high sequencing availability according to the WHO’s Global genomic surveillance strategic objectives for reinforcing the capacities that include sample collection, diagnostics, data sharing, and analysis [30]. This is consistent with the observed increase in shared viral sequences in GISAID [31]. The continuous monitoring and data analysis through the RELECOV network offered a comprehensive knowledge about the virus dynamics and its circulating variants at a national level, increasing this knowledge in Europe and in the European/trans-national dimension.

The benefit of the SARS-CoV-2 genomic surveillance includes the tracking of changes in the viral genome and predicting their implications. Recently published studies have shown the impact of mutations in viral infectivity and immunogenicity [32,33,34,35,36]. The Omicron variant has been the variant with the largest number of mutations described in the *S* gene. A recently published study showed ten exclusive mutations found for BA.1 and BA.2 and only one mutation in the *S* gene was shared with the Delta variant [37]. However, we observed that nine mutations in Omicron lineages and sublineages were present in all of them. Furthermore, only five lineage-defining mutations across the viral genome are shared by Omicron and Delta variants in more than 95% of lineages and sublineages of both variants. Additionally, by focusing on the *S* gene, the Omicron variant has six mutations present in all lineages/sublineages, suggesting that these positions could have an important role in the establishment of this variant worldwide, , reinforcing the need for viral genomic surveillance due to the central role of the spike protein as a target for vaccine design.

Viral genomic surveillance is an essential tool, but the pillars of a molecular surveillance approach require good quality data (sequence and metadata). The quality control assessment is a perfect procedure to determine the performance of individual laboratories, identify procedural issues, establish the effectiveness and intercomparability of the methods used, and identify interlaboratory differences that can provide additional confidence to laboratories for the characterization of SARS-CoV-2. Our results for the QCA showed the inherent complexity to obtain comparable results regarding the lineage versus variant assignment, which would make monitoring difficult in the context of public health. Until July 2022, a totally of 2175 different lineages has been assigned to SARS-CoV-2 using the Pangolin tool [9]. Of these, 212 have been reassigned and some of them have even been eliminated [38]. This fact, in addition to the difficulties in obtaining the whole viral sequence derived from sample and technical issues, increases the SARS-CoV-2 monitoring complexity in the public health context. Even if we consider the success rate for obtaining a high-coverage complete sequence according to GISAID (sequences with less than 1% of undefined bases (NNNs) and insertions and/or deletions verified by the submitter), regarding the worldwide data available from GISAID (queried on 2022.10.16), only 42.4% (5,743,076/13,551,277) of the deposited sequences can be considered whole-genome sequences with high coverage. Furthermore, this does not guarantee the quality in the genomic regions of great interest such as the *S* gene for sequences with high coverage. In this situation, the establishment of clear criteria based on viral biological characteristics and their impact on human health leading to a consensus nomenclature is required for surveillance at the public health level.

In view of these difficulties, the use of tools for the phylogenetic analysis quickly provides knowledge about the viral molecular evolution reflected in clusters involving molecular changes that could be associated with biological features relevant for viral surveillance in the public health system. In this sense, the nomenclature proposed by the WHO takes on more relevance [20]. The phylogenetic analyses carried out from the complete viral genome and the *S* gene generated similar results highlighting the relevance of the *S* gene sequence variation in the public health context. This could be explained because the SARS-CoV-2 *S* gene presents the highest non-synonymous mutation rates in comparison to the rest of the viral genome [12], as well as their role in the SARS-CoV-2 environmental adaptation [39]. On the other hand, viral surface proteins are highly exposed to pressure by the immune system, leading to more frequent antigenic drift [40]. Since this region is also the target for vaccine design, this makes it a relevant issue for public health surveillance. Thus, although the availability of more information on the viral genome is important, it is more important to obtain this information with higher quality and focus on what may have a direct impact on health. In this sense, the selection of specific viral genome regions to perform phylogenetic analysis have been carried out for a long time in regard to public health surveillance, as is the case with the *Influenza* virus, where hemagglutinin is the most relevant region for its surveillance [41].

In summary, the establishment of molecular nationwide surveillance networks are essential for addressing science-based public health decisions and for achieving control of infectious diseases, and this should be a priority worldwide. The availability of methodologies for the sequencing of the whole viral genome has been a great advance, but we must not forget that other technologies that require fewer resources can also be used to obtain molecular information useful for surveillance and evaluation of vaccine effectiveness. Future actions should be aimed to ensuring high-quality data, focusing on relevant data for public heath applications and quality–cost evaluations that could favour the inclusion of surveillance in low- and middle-income countries, taking into account the global disparities on sequencing capacity [42] in order to increase the knowledge that is relevant to public health.

## 4. Materials and Methods

### 4.1. SARS-CoV-2 Viral Sequences Deposited in GISAID by the RELECOV Network Members

To assess the kick-off activity of the RELECOV network, we studied the level of viral sequence data sharing from Spain samples through GISAID. To disentangle the spectrum of variants circulating over time during the first 6 months of network activity, we established three periods for evaluation: a first quarter between 05.09.2021 and 05.12.2021; a second quarter between 06.12.2021and 08.03.2022; and a semi-annual period comprising both quarters. For this purpose, the sequences available in GISAID from Spain and deposited by RELECOV network members during this period were retrieved. A total of 20,675 viral sequences were deposited in GISAID during the first quarter from Spain, of which 20,435 sequences were deposited by 29 RELECOV network members. Second quarter data comprised 28,151 viral sequences from Spain, of which 28,143 corresponded to sequences deposited by 29 RELECOV network members. Finally, the semi-annual data analyses covered both quarters with a total of 48,578 viral genomes that were deposited in GISAID by 30 RELECOV network members.

### 4.2. Tracking the Changes in the SARS-CoV-2 Delta and Omicron Variants

Delta and Omicron variants were selected considering their prevalence from 03.09.2021 to 04.11.2022. A database containing information on lineage-defining mutations was generated for all lineages and sublineages of Delta and Omicron variants using information available in the outbreak.info platform [21]. We updated and reviewed the database every two weeks due to the changes that occur in the lineage assignments and lineage-defining mutations. A total of 441 lineages and sublineages were included in the study: 248 corresponding to Delta and 205 to the Omicron variant (Supplementary File S3 and Supplementary Table S1).

### 4.3. Quality Control Assessment (QCA) of SARS-CoV-2 Viral Genome Sequencing

A QCA of SARS-CoV-2 sequencing and characterization was organized and prepared at the National Center of Microbiology. This QCA was performed between 22.12.2021 and 28.01.2022 in order to evaluate the sequencing capacity of the RELECOV network laboratories and the skills in the bioinformatic assignment of variants and lineages of SARS-CoV-2.

The QCA included ten lyophilized RNA samples of different SARS-CoV-2 viruses isolated from VERO E6 cell cultures. The panel was made up of eight SARS-CoV-2 samples belonging to VOCs, one variant of interest (VOI) and another sample corresponding to the A.28 lineage not classified by the WHO as a VOC, VOI, or Variant Under Monitoring (VUM). The samples included in the QCA were selected based on their relevance in terms of virus circulation in Spain. The preparation of samples, the homogeneity prior to distribution, and the stability and sequencing tests of each virus were carried out at the National Centre of Microbiology and at the central scientific–technical units, the Genomics unit, and the Bioinformatics unit from the Instituto de Salud Carlos III. The characteristics of the panel and the evaluation of homogeneity and stability are shown in Table 4 and Table 5, respectively.

A total of 15 library preparation kits were used by the participants in this panel: ABL DeepChek^®^ Assay WG SC2 V1 (Advanced Biological Laboratories, Luxembourg), Genexus™ Library Strips 1 and 2-AS; Ion AmpliSeq Chef DL8 Kit, Ion Ampliseq Library Kit and Ion AmpliSeq™ SARS-CoV-2 Insight Research Assay-GS Manual (Thermo Fisher Scientific Inc, Waltham, MA, USA); Illumina COVIDseq Kit, Illumina DNA Prep Tagmentation, Nextera DNA Flex, Nextera XT (Illumina Inc. San Diego, CA, USA) NEBNext^®^ ARTIC SARS-CoV-2 FS Library Prep Kit, NEBNext^®^ Fast DNA Library Prep Set for Ion Torrent™, NEBNext^®^ Ultra™ II FS DNA Library Prep Kit for Illumina (New England Biolabs, UK); Oxford Nanopore Sequencing Kit (Oxford Nanopore Technologies, Oxford, UK); and ViroKey SQ FLEX Library Prep Reagents (Vela Diagnostics, The Kendall, Singapur). Ten sequencing systems were used including: Illumina iSeq 100, Illumina MiSeq, Illumina NextSeq, Illumina NovaSeq 6000 (Illumina Inc. San Diego, CA, USA); Ion Torrent, Ion Torrent PGM, Ion Torrent S5, Ion Torrent S5 XL, Ion Torrent Genexus™ Integrated Sequencer (Ion Torrent Systems, Inc., Gilford, NH, USA); and MinION (Oxford Nanopore Technologies, Oxford, UK).

### 4.4. Phylogenetic Analysis of SARS-CoV-2 Viral Genome Sequences

A total of 2557 worldwide viral sequences corresponding to different lineages corresponding to Alpha, Beta, Gamma, Delta, Eta, Iota, Kappa, Lambda, Mu, Theta, and Omicron were retrieved from GISAID. Reference sequences used in the phylogenetic analysis must agree the following criteria to be included:

- Correspondence to a specific variant described by the WHO.
- First described sequences with complete viral genome, high coverage (according to GISAID those sequences with less than 1% of undefined bases (NNNs) and insertions and/or deletions verified by the submitter), and complete collection date.
- At least 3–6 sequences per lineage and/or sublineage described for each variant.
- Does not present unresolved nucleotide positions.
- Size of the fragment between position 55 and 29,674 (ORF10 end) refers to the reference sequence NC_04552 available in NCBI [43]

We used MEGA7 [43] and Bioedit 7.2.5 programs [44,45] in order to ensure compliance with established criteria and to achieve a working matrix with good quality for all retrieved sequences. Finally, a total of 714 sequences were included into the final matrix to obtain a reference tree. The list of selected sequences and related data are available on GISAID and in Supplementary File S1. Omicron lineage BA.3 was not included since no sequence was found meeting all the criteria. The final matrix of selected sequences was aligned using the online service of MAFFT version 7 [46]. The alignment was reviewed and corrected to ensure virus genome biological sense since some nucleotide positions were found that did not meet this criterion. Secondly, we also generate a matrix containing only the *S* gene fragment (3821 nt) to compare the resolution of both phylogenetic analyses. Subsequently, phylogenetic analysis for the complete genome and the *S* gene were performed using IQ-TREE 2 [23] and FastTree [22], and resources available from the CIPRES Science Gateway [47]. We inferred a maximum-likelihood tree using the GTR+G model, combining the SH-aLRT test and ultrafast bootstrap with 1000 replicates in IQ-TREE 2, and the fastest GTR+G model and 1000 replicates in FastTree.

## 5. Conclusions

The RELECOV network actions to date have made it possible to improve and enhance the surveillance of the SARS-CoV-2 viruses that circulate in Spain through sequences analysis, monitoring of virus genome changes, and a quality control assessment of the sequencing at the national level. In addition, the phylogenetic analysis to characterize SARS-CoV-2 allowed us to gain knowledge about the circulation of variants in Spain in an independent and rapid manner in order to assist public health decisions. The described actions in the RELECOV network have comprised efforts directed to control the quality of data obtained and shared, and overall, to understand the data that could provide meaningful biological interpretation to focus on viral molecular surveillance applications for the public health.

## Figures and Tables

**Table 1:** SARS-CoV-2 viral sequences from Spain deposited in GISAID between 05.09.2021 and 05.12.2021.

Variant	Number of Sequences	Number of Lineages
Alpha	1185	3
Beta	87	2
Gamma	96	9
Delta	18,272	126
Mu	171	3
Omicron	8	4
Lambda	40	2
^2^ VUM	1	1
Eta	27	1
Epsilon	1	1
^1^ NA	483	48
Unassigned lineage	61	NA
Recombinant	3	NA
Total	20,435	200

^1^ NA: Not applicable (lineages without variant assignment). ^2^ VUM: Variant Under Monitoring.

**Table 2:** SARS-CoV-2 viral sequences from Spain deposited in GISAID between 06.12.2021 and 08.03.2022.

Variant	Number of Sequences	Number of Lineages
Alpha	72	2
Beta	2	2
Delta	12,733	107
Gamma	4	2
Mu	15	3
Omicron	15,251	48
Unassigned lineage	16	NA
Recombinant	1	NA
^1^ NA	49	8
Total	28,143	172

^1^ NA: Not applicable (lineages without variant assignment).

**Table 3:** Characteristics of the samples and results of the 37 RELECOV network participants reporting results in the QCA.

Sample	Variant	Lineage	Variant	Lineage
			Number of Correct Results (%)	Number of Correct Results (%)
#1	Alpha	B.1.1.7	37 (100)	36 (97.30)
#2	Beta	B.1.351	37 (100)	37 (100)
#3	^1^ NA	A.28	36 (97.30)	36 (97.30)
#4	Mu	B.1.621	37 (100)	37 (100)
#5	Gamma	P.1	37 (100)	34 (91.89)
#6	Delta	AY.9.2	37 (100)	33 (89.19)
#7	Delta	AY.43	14 (37.83)	11 (29.73)
#8	Delta	AY.94	36 (97.30)	31 (83.78)
#9	Delta	AY.94	37 (100)	32 (86.49)
#10	Delta	AY.43	37 (100)	37 (100)

^1^ NA: Not applicable.

**Table 4:** Characteristics of the QCA panel of samples.

Sample	Variant	Lineages	Pre-Shipment ^2^ Cts	Post-Shipment ^2^ Cts	
				56 Days	96 Days
#1	Alpha	B.1.1.7	19.1	17.8	21.0
#2	Beta	B.1.351	17.6	16.0	19.2
#3	^1^ NA	A.28	22.8	19.6	21.6
#4	Mu	B.1.621	18.7	17.0	18.5
#5	Gamma	P.1	17.7	18.7	20.6
#6	Delta	AY.9.2	25.2	22.6	25.7
#7	Delta	AY.43	21.3	21.6	24.7
#8	Delta	AY.94	16.2	16.6	19.4
#9	Delta	AY.94	16.2	19.4	20.5
#10	Delta	AY.43	21.3	20.8	21.9

^1^ NA: Not applicable; ^2^ Cts = Cycle threshold.

**Table 5:** Sample homogeneity and stability.

Sample	Average	^1^ SD	^2^ CI	Average ± CI ^2^ (95%)
#1	19.3	1.33	1.50	19.3 ± 1.50
#2	17.6	1.31	1.48	17.6 ± 1.48
#3	21.3	1.33	1.50	21.3 ± 1.50
#4	18.1	0.77	0.87	18.1 ± 0.87
#5	19.0	1.21	1.36	19.0 ± 1.36
#6	24.5	1.34	1.52	24.5 ± 1.52
#7	22.6	1.52	1.72	22.6 ± 1.72
#8	17.4	1.44	1.63	17.4 ± 1.63
#9	18.7	1.83	2.07	18.7 ± 2.07
#10	21.3	0.46	0.52	21.3 ± 0.52

**^1^** SD: standard deviation; ^2^ CI: confidence interval.

## Data Availability

Publicly available datasets were analysed in this study. This data can be found here: https://gisaid.org/ (accessed on 1 March 2023) and https://outbreak.info/ (accessed on 1 October 2022).

## Supplementary Materials

The supporting information can be downloaded at: https://www.mdpi.com/article/10.3390/ijms24108573/s1.
