# Supplementary material for: Enhancing SARS-CoV-2 Surveillance through Regular Genomic Sequencing in Spain: The RELECOV Network
Source: Int J Mol Sci. 2023 May 10;24(10):8573. doi: 10.3390/ijms24108573 (PMC10218691; doi:10.3390/ijms24108573)
Supplement: Supplementary file 1 [file ijms-24-08573-s001.zip › Supplementary File S1.Gisaid_supplemental_table_epi_set_230301uh.pdf]

Data Availability

GISAID Identifier:	EPI_SET_230301uh
doi:	10.55876/gis8.230301uh

All genome sequences and associated metadata in this dataset are published in GISAID's EpiCoV database. To view the contributors of each individual sequence with details such as accession number, Virus name, Collection date, Originating Lab and Submitting Lab and the list of Authors, visit 10.55876/gis8.230301uh

Data Snapshot

- EPI_SET_230301uh is composed of 713 individual genome sequences.
- The collection dates range from 2020-03-06 to 2022-05-12;
- Data were collected in 76 countries and territories;
- All sequences in this dataset are compared relative to hCoV-19/Wuhan/WIV04/2019 (WIV04), the official reference sequence employed by GISAID (EPI_ISL_402124). Learn more at https://gisaid.org/WIV04.
