# Supplementary material for: Enhancing SARS-CoV-2 Surveillance through Regular Genomic Sequencing in Spain: The RELECOV Network
Source: Int J Mol Sci. 2023 May 10;24(10):8573. doi: 10.3390/ijms24108573 (PMC10218691; doi:10.3390/ijms24108573)
Supplement: Supplementary file 1 [file ijms-24-08573-s001.zip › Supplementary File S2. RELECOV network members.pdf]

RELECOV network members	Affiliation
Adolfo de Salazar; Carlos S. Casimiro-Soriguer	Hospital Universitario San Cecilio, Granada. CIBER de Enfermedades Infecciosas (CIBERINFEC), Instituto de Salud Carlos III; Computational Medicine Platform, Andalusian Public Foundation Progress and Health-FPS. Institute of Biomedicine of Seville, IBiS, University Hospital Virgen del Rocío, CSIC, University of Sevilla
Javier Perez-Florido, Joaquin Dopazo	Hospital Universitario Virgen del Rocio. Sevilla; Computational Medicine Platform, Andalusian Public Foundation Progress and Health-FPS. Institute of Biomedicine of Seville, IBiS, University Hospital Virgen del Rocío, CSIC, University of Sevilla
Jessica Bueno, Sonia Algarate	Hospital Clínico Universitario Lozano Blesa
Antonio Rezusta; Ana Milagro Beamonte	Hospital Universitario Miguel Servet
Alba de Martino; Mark Strunk	Centro de Investigación Biomédica de Aragón
José Antonio Boga; Zulema Pérez Martínez.	Hospital Universitario Central de Asturias
Antonio Oliver; Gabriel Cabot	Hospital Universitario Son Espases
Diego García-Martínez de Artola; Helena Gil-Campesino	Hospital Universitario Ntra. Sra de Candelaria
Jose M. Lorenzo-Salazar; Rafaela González-Montelongo	Instituto Tecnológico y de Energías Renovables, S.A.
Ana Bordes; Francisco Chamizo López	Hospital Universitario de Gran Canaria Dr. Negrín
Jesus Rodriguez-Lozano; Jorge Calvo-Montes	Hospital Universitario Marqués de Valdecilla-IDIVAL. CIBER de Enfermedades Infecciosas (CIBERINFEC), Instituto de Salud Carlos III
Soledad Illescas; José Martínez Alarcón	Hospital Universitario de Ciudad Real
Eva Heredero Gálvez; Patricia Trevisi	Complejo Hospitalario de Toledo.
Caridad Sainz de Baranda; Lorena Robles Fonseca	Complejo Hospitalario Universitario de Albacete
Jose Maria Eiros; Marta Hernández; David Abad; Silvia Rojo; Sonsoles Garcinuño; Gabriel March	Consorcio LUCIA (SACYL, ITACYL, UBU, UVa)
Andrés Antón; Cristina Andrés	Hospital Universitari Vall d’Hebron
Ignacio Blanco Guillermo; Elisa Martró	Hospital Universitari Germans Trias i Pujol

Mª Ángeles Marcos Maeso; Elisa Rubio	Hospital Clínic de Barcelona
Jordi Càmara Mas; Sara Martí	Hospital Universitari de Bellvitge-IDIBELL-CIBERes
Teresa Sans Mateu; Gemma Recio Comí	Hospital Joan XXIII
Mireia Canal; Juanjo Hernández	Laboratori de Referencia de Cataluya
Glòria Soria; Francisco Vidal	Banc de Sang i Teixits Catalunya
Mel·lina Pinsach; Ramon Brugada	Hospital Dr. Josep Trueta
Salomé Hijano Villegas; Patricia González Donapetry	Hospital Universitario de Ceuta
María del Carmen González Velasco; Cristina Eugenia Gaona Álvarez	Hospital Universitario de Badajoz
German Bou Arevalo; Ana Fernandez Gonzalez	Hospital de A Coruña
Maria Luisa Perez del Molino; José Javier Costa Alcalde	Complejo Hospitralario Santiago de Compostela
Benito Regueiro Garcia; Carlos Daviña Núñez	Complexo Hospitalario Universitario de Vigo
María Pilar Bea-Escudero	Plataforma de Genómica y Bioinformática. La Rioja
Manuel Ponce-Alonso; Laura Martínez García	Hospital Universitario Ramón y Cajal
Esther Viedma; Carmen Martin-Higuera	Hospital Universitario 12 de Octubre. Biomedical Research Institute imas12
Julio García Rodríguez; Iván Bloise Sánchez	Hospital Universitario La Paz
Patricia Muñoz; Laura Perez Lago	Hospital General Universitario Gregorio Marañón
Sergio Román Soto; Víctor Céspedes Balderrama	Hospital Comarcal de Melilla
Laura Moreno Parrado; Marina Simón Páez	Hospital Universitario Virgen de la Arrixaca
Ana Navascues; Miguel Fernández Huerta	Hospital Universitario de Navarra
Mikel Urrutikoetxea Gutierrez	Hospital Universitario de Basurto
Mikel Gallego Rodrigo; Clara Lejarraga Cañas	Hospital Universitario Cruces
Ane Sorarrain Zarraga; Milagrosa Montes	Hospital Universitario Donostia
Sara Monzón Fernández; Sarai Varona Fernández	Unidad de Bioinformática. Instituto de Salud Carlos III

María Alma Bracho Lapiedra; Lidia Ruiz Roldán	FISABIO Univ.Valencia-Epidemiología Molecular
Nieves Gonzalo Jimenez; Antonio Galiana	Hospital General Universitario de Elche
Concepción Gimeno; Rafael Medina	Consorcio Hospital General Universitario de Valencia
Juan Carlos Rodríguez Díaz; Maria Paz Ventero	Hospital General Universitario de Alicante
David Navarro; Eliseo Albert	Hospital Clínico Universitario de Valencia
Dolores Tirado; Marta Gil	Hospital General Universitario de Castellón
José Miguel Nogueira; Juan Alberola	Hospital Doctor Peset de Valencia
José Luis López Hontangas; Luis Ramón	Hospital Universitario y Politécnico La Fe de Valencia
