# Supplementary material for: Enhancing SARS-CoV-2 Surveillance through Regular Genomic Sequencing in Spain: The RELECOV Network
Source: Int J Mol Sci. 2023 May 10;24(10):8573. doi: 10.3390/ijms24108573 (PMC10218691; doi:10.3390/ijms24108573)

Supplementary Table S1. Detailed Lineages and sublineages included in this study for viral tracking changes in SARS-CoV-2.

**DELTA VARIANT**	**OMICRON VARIANT**
Lineage	Lineage
AY.1	BA.1
AY.10	BA.1.1
AY.11	BA.2
AY.12	BA.3
AY.13	BA.1.1.1
AY.14	BA.1.1.2
AY.15	BA.1.1.3
AY.16	BA.1.1.4
AY.17	BA.1.1.5
AY.18	BA.1.1.6
AY.19	BA.1.1.7
AY.2	BA.1.1.8
AY.20	BA.1.1.9
AY.21	BA.1.1.10
AY.22	BA.1.1.11
AY.23	BA.1.1.12
AY.23.1	BA.1.1.13
AY.24	BA.1.1.14
AY.25	BA.1.1.15
AY.26	BA.1.1.16
AY.27	BA.1.1.17
AY.28	BA.1.2
AY.29	BA.1.3
AY.29.1	BA.1.4
AY.3	BA.1.5
AY.3.1	BA.1.6
AY.30	BA.1.7
AY.31	BA.1.8
AY.32	BA.1.9
AY.33	BA.1.10
AY.34	BA.1.12
AY.35	BA.1.13

AY.36	BA.1.13.1
AY.37	BA.1.14
AY.38	BA.1.14.1
AY.39	BA.1.14.2
AY.39.1	BA.1.15
AY.39.1.1	BA.1.15.1
AY.4	BA.1.15.2
AY.4.1	BA.1.16
AY.4.2	BA.1.16.1
AY.4.4	BA.1.16.2
AY.4.5	BA.1.17
AY.40	BA.1.17.1
AY.41	BA.1.17.2
AY.5	BA.1.18
AY.5.1	BA.1.19
AY.5.2	BA.1.20
AY.6	BA.1.21
AY.7	BA.1.21.1
AY.7.1	BA.2.1
AY.7.2	BA.2.2
AY.8	BA.2.3
AY.9	BA.2.3.1
B.1.617.2	BA.2.3.2
AY.9.1	BA.2.4
AY.9.2	BA.2.5
AY.9.2.1	BA.2.6
AY.16.1	BA.2.7
AY.34.1	BA.2.8
AY.39.2	BA.2.9
AY.42	BA.2.9.1
AY.43	BA.2.10
AY.44	BA.2.10.1
AY.45	BA.2.11
AY.46	BA.2.12
AY.46.1	BA.2.12.1
AY.46.2	BA.2.13
AY.46.3	BA.2.14

AY.46.4	BA.2.15
AY.46.5	BA.2.16
AY.46.6	BA.4
AY.47	BA.5
AY.48	BA.1.1.18
AY.49	BA.1.22
AY.50	BA.2.3.4
AY.51	BA.2.9.2
AY.52	BA.2.10.2
AY.53	BA.2.17
AY.54	BA.2.18
AY.55	BA.2.19
AY.56	BA.2.20
AY.57	BA.2.21
AY.58	BA.2.22
AY.59	BA.2.23
AY.60	BA.2.23.1
AY.61	BA.2.24
AY.62	BA.2.25
AY.63	BA.2.25.1
AY.64	BA.2.26
AY.65	BA.2.27
AY.66	BA.2.28
AY.67	BA.2.29
AY.68	BA.2.30
AY.69	BA.2.31
AY.70	BA.2.32
AY.71	BA.2.33
AY.72	BA.2.34
AY.73	BA.2.35
AY.74	BA.2.36
AY.75	BA.2.37
AY.75.1	BA.2.38
AY.76	BA.2.39
AY.77	BA.2.40
AY.78	BA.2.40.1
AY.79	BA.2.41

AY.80	BA.3.1
AY.81	BA.5.1
AY.82	BC.1
AY.83	BC.2
AY.84	BD.1
AY.85	BE.1
AY.86	BE.1.1
AY.87	BE.2
AY.88	BA.1.15.3
AY.89	BA.1.23
AY.90	BA.1.24
AY.91	BA.2.2.1
AY.91.1	BA.2.3.5
AY.92	BA.2.3.6
AY.93	BA.2.3.7
AY.94	BA.2.3.8
AY.95	BA.2.3.9
AY.96	BA.2.3.10
AY.97	BA.2.3.11
AY.98	BA.2.3.12
AY.98.1	BA.2.3.13
AY.99	BA.2.3.14
AY.99.1	BA.2.3.15
AY.99.2	BA.2.3.16
AY.100	BA.2.3.17
AY.101	BA.2.3.18
AY.102	BA.2.9.3
AY.103	BA.2.9.4
AY.104	BA.2.9.5
AY.105	BA.2.10.3
AY.106	BA.2.12.2
AY.107	BA.2.38.1
AY.108	BA.2.42
AY.109	BA.2.43
AY.110	BA.2.44
AY.111	BA.2.45
AY.112	BA.2.46

AY.113	BA.2.47
AY.114	BA.2.48
AY.116	BA.2.49
AY.116.1	BA.2.50
AY.117	BA.2.51
AY.3.2	BA.2.52
AY.3.3	BA.2.53
AY.4.2.1	BA.2.54
AY.4.2.2	BA.2.55
AY.4.2.3	BA.2.56
AY.4.6	BA.2.56.1
AY.4.7	BA.2.57
AY.4.8	BA.2.58
AY.4.9	BA.2.59
AY.4.10	BA.2.60
AY.5.3	BA.2.61
AY.5.4	BA.2.62
AY.5.5	BA.2.63
AY.9.2.2	BA.2.64
AY.20.1	BA.2.65
AY.23.2	BA.2.66
AY.25.1	BA.2.67
AY.25.1.1	BA.2.68
AY.26.1	BA.2.69
AY.33.1	BA.2.70
AY.34.1.1	BA.2.71
AY.34.2	BA.2.72
AY.39.1.2	BA.2.73
AY.39.1.3	BA.2.74
AY.42.1	BA.2.75
AY.43.1	BA.2.76
AY.43.2	BA.2.77
AY.43.3	BA.2.80
AY.43.4	BA.2.81
AY.43.5	BA.4.1
AY.43.6	BA.4.1.1
AY.43.7	BA.4.1.2

AY.46.6.1
AY.75.2
AY.75.3
AY.102.1
AY.102.2
AY.112.1
AY.118
AY.119
AY.119.1
AY.119.2
AY.120
AY.120.1
AY.120.2
AY.120.2.1
AY.121
AY.121.1
AY.122
AY.122.1
AY.122.2
AY.122.3
AY.123
AY.123.1
AY.124
AY.124.1
AY.125
AY.126
AY.127
AY.127.1
AY.128
AY.129
AY.131
AY.132
AY.133
AY.3.4
AY.4.2.4
AY.4.11
AY.4.12

BA.4.1.3
BA.4.1.4
BA.4.2
BA.4.3
BA.4.4
BA.4.5
BA.4.6
BA.4.7
BA.5.1.1
BA.5.1.2
BA.5.1.3
BA.5.1.4
BA.5.2
BA.5.2.1
BA.5.2.2
BA.5.2.3
BA.5.2.4
BA.5.3
BA.5.3.1
BA.5.3.2
BA.5.3.3
BA.5.3.4
BA.5.5
BA.5.6
BE.3

AY.25.3
AY.103.1
AY.103.2
AY.122.4
AY.125.1
AY.4.2.5
AY.5.6
AY.5.7
AY.25.1.2
AY.25.2
AY.29.2
AY.33.2
AY.36.1
AY.39.3
AY.43.8
AY.43.9
AY.112.2
AY.112.3
AY.122.5
AY.122.6
AY.124.1.1
AY.127.2
AY.127.3
AY.4.13
AY.4.14
AY.4.15
AY.4.16
AY.4.17
AY.134
AY.4.3
AY.24.1

Supplementary Figure S1. Timecourse of variant distribution by month in all sumited sequences to GISAID until 2022-03-15. Source: GISAID_hCoV-19_Analysis_Update_20220315.

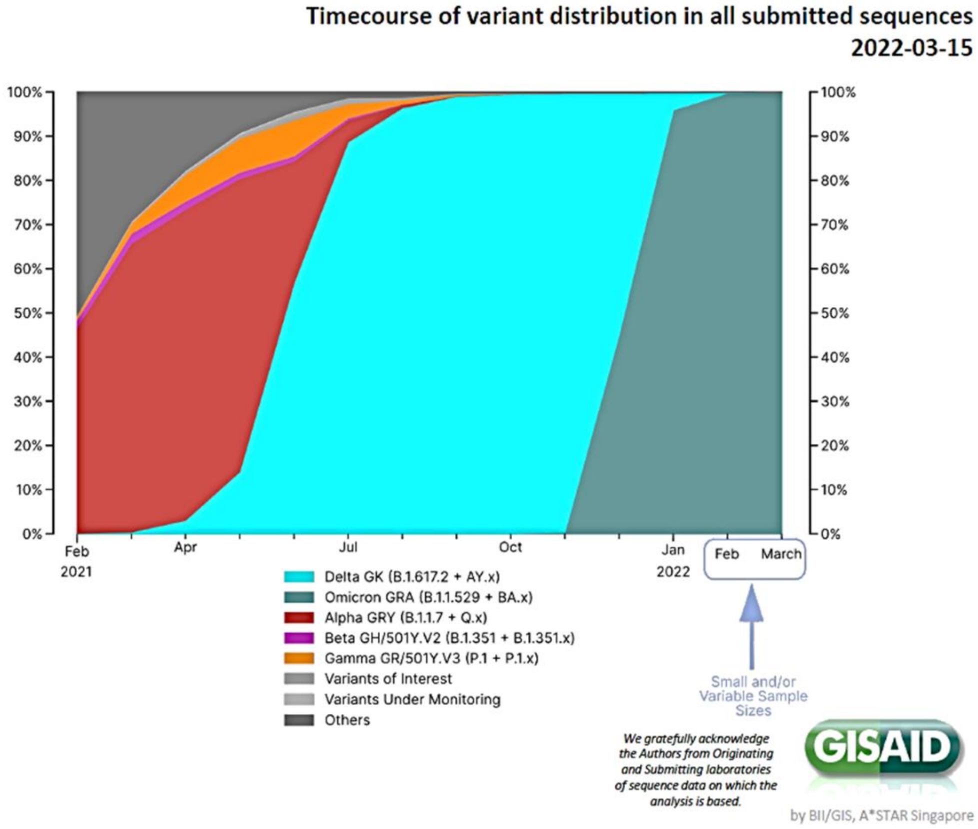

Supplement: Supplementary file 1 [file ijms-24-08573-s001.zip › Supplementary File S3.pdf]
